# High Entrepreneurship, Leadership, and Professionalism (HELP): Toward an Integrated, Empirically Based Perspective

**DOI:** 10.3389/fpsyg.2016.01842

**Published:** 2016-11-25

**Authors:** Annamaria Di Fabio, Ornella Bucci, Alessio Gori

**Affiliations:** Department of Education and Psychology (Psychology Section), University of FlorenceFlorence, Italy

**Keywords:** entrepreneurship, leadership, professionalism, psychometric properties, healthy business, healthy organization

## Abstract

This article evaluates the psychometric properties of a new measure for assessing the constructs of entrepreneurship, leadership, and professionalism, from an integrated point of view, the High Entrepreneurship, Leadership and Professionalism Questionnaire (HELP-Q). Exploratory factor analysis indicated a factor structure with three principal dimensions, and confirmatory factor analysis and goodness of fit indices indicated a good fit of the model to the data. All the dimensions showed good values of internal consistency. The results of the study thus indicate that the HELP-Q is a short and easily administered instrument with good psychometric properties that can promote entrepreneurship, leadership, and professionalism in workers as well as in those who are preparing to enter the turbulent 21st century labor market.

## Introduction

In today’s rapidly changing world of work, a career is more than just a vocation or occupation—it is about creating meaning from an unfolding set of experiences and the lessons of a lifetime (Super, 1957, 1980; Savickas, 2002; Chan et al., 2012). People need specific personality characteristics and talents to achieve their career goals (Di Fabio and Maree, 2012; Di Fabio and Saklofske, 2014a,b; Di Fabio and Kenny, 2015, 2016a,b; Di Fabio, 2016a; Di Fabio et al., 2016b), and three constructs considered crucial in career management are entrepreneurship, leadership, and professionalism (Shane and Venkataraman, 2000; Kuratko, 2005; Chan et al., 2012).

These constructs have been studied separately in the past, and only recently attention has been given to studying them together (Chan et al., 2012; Renko et al., 2012). The major focus has been on the construct entrepreneurial leadership (Renko et al., 2012) while attempts to integrate it with the construct professionalism have only just begun.

This article sets out to review existing research on entrepreneurial leadership and professionalism and to present the key elements of these constructs. After describing the domains of entrepreneurship, leadership, and professionalism, we analyze the construct entrepreneurial leadership and suggest how it too can be integrated with the construct professionalism. We then propose and empirically test a scale to measure entrepreneurship, leadership, and professionalism from an integrated point of view in terms of career construction and conclude with the implications for future research and managerial practice.

### Entrepreneurship

Entrepreneurial behaviors are becoming increasingly important in a variety of contexts. In organizations, these behaviors foster innovation and adaptation to changing environments (Renko et al., 2015). Entrepreneurial behavior is described as the processes, practices, and decision-making activities that lead to entrepreneurship (Lumpkin and Dess, 1996). According to Lumpkin and Dess (1996), key entrepreneurial processes are autonomy, innovativeness, risk-taking, proactiveness, and competitive aggressiveness.

Autonomy refers to the independent action of a person in carrying an idea or a vision through to completion. Innovativeness is the tendency to engage with new ideas and to conduct experimentation that may result in new products. Proactiveness implies acting in anticipation of future problems, needs, or changes. Competitive aggressiveness involves directly and intensely challenging competitors. Effective entrepreneurial education should facilitate and promote these entrepreneurial behaviors (Okudana and Rzasa, 2006).

Regarding entrepreneurship, two different academic approaches can be seen in the literature: the study of “entrepreneurial traits” and the study of “entrepreneurial rates.” Entrepreneurial traits research (a major division of the empirical work in the field) focuses on the individual differences between entrepreneurs while entrepreneurial rates research examines environmental influences (often economic conditions) on the propensity to start a business or to innovate. By and large, the rates approach ignores the constructs used by traits researchers, and the traits approach does not incorporate the constructs used by rates researchers. Rates researchers are more likely to be interested in studying changes in the rate of the establishment of new businesses over time and consequently more commonly make use of longitudinal research designs (Shane, 1996; Vecchio, 2003). Traits researchers, while acknowledging longitudinal research, more typically adopt cross-sectional designs as part of a survey-based approach. Because of these differences in focus and preferred study design, the two approaches are not particularly supplementary to one another. Instead, each approach fosters a knowledge base that is cumulative within itself and is not amenable to integration with other approaches. The distinction between the traits and rates approaches can be best understood as a distinction between a supply side approach and a demand-side approach (Thornton, 1999). The supply side approach focuses on the propensity and availability of individuals for entrepreneurial roles while the demand-side approach focuses on the number and nature of entrepreneurial roles that need to be filled. While entrepreneurship requires an attitude toward risk taking that includes using one’s instinctive feelings, many aspects of entrepreneurship can also be taught (Garavan and O’Cinneide, 1994).

### Leadership

Leadership, in general, involves influencing the activities of an organized group toward goal achievement (Rauch and Behling, 1984, p. 46; House et al., 1999, p. 184; Boyatzis, 2006). It differs from management, which tends to be focused on coordination and planning (Zaleznik, 1977; Michael et al., 2002; Renko et al., 2015).

Academic authors have presented a range of views on gender differences and similarities in different leadership styles (e.g., Eagly and Johnson, 1990) such as transformational, transactional, and laissez-faire leadership styles (Eagly et al., 2003). Burns (1978) introduced transformational leadership theory, which was further developed by Bass and Avolio (1994). According to these authors, transformational leadership has four components: charismatic role modeling, individualized consideration, inspirational motivation, and intellectual stimulation. Using charisma, the leader inspires admiration, respect, and loyalty and emphasizes the importance of having a collective sense of mission. Through individualized consideration, the leader builds a one-on-one relationship with his or her followers and understands and takes into consideration their differing needs, skills, and aspirations (Gumusluoglu and Ilsev, 2009). Through inspirational motivation, the leader articulates an exciting vision of the future, shows his or her followers how to achieve the particular goals, and expresses his or her belief that they can achieve the goals (Gumusluoglu and Ilsev, 2009). Through intellectual stimulation, the leader broadens and elevates the interests of his or her followers (Bass, 1990) and encourages them to think about old problems in new ways (Bass, 1985).

Transformational leadership behaviors closely match the determinants of innovation and creativity in the workplace, some of which are vision, support for innovation, autonomy, encouragement, recognition, and challenge (Elkins and Keller, 2003; Gumusluoglu and Ilsev, 2009). Burns (1978) and other researchers (see Bass, 1998; Avolio, 1999) compare transformational leaders to transactional leaders, who appeal to followers’ self-interest by establishing exchange relationships with them (i.e., where ideas and resources are exchanged between leaders and followers) (Eagly et al., 2003). This type of leadership involves managing in the more conventional sense of clarifying subordinates’ responsibilities, rewarding them for meeting objectives, and correcting them for failing to meet objectives. Although there are thus two different styles of leadership—transformational and transactional—both are displayed by effective leaders. In addition to these two styles, the above authors distinguish a laissez-faire style marked by a general failure to take responsibility for managing (Eagly et al., 2003).

The new approaches to the study of leadership acknowledge several styles of leadership: sustainable leadership, servant leadership, authentic leadership, ethical leadership, mindful leadership, benevolent leadership, and decent leadership (Di Fabio, in press), each of which has peculiar characteristics. For example, sustainable leadership refers to the shared responsibility not to deplete human or financial resources unduly and not to cause social or environmental damage (Hargreaves et al., 2003; Hargreaves and Fink, 2004); servant leadership considers the personal growth of subordinates as paramount and more important than achieving the objectives of the company or of the leaders (Ehrhart, 2004); authentic leadership refers to a style of leadership and ethics where the emphasis is more on subordinates’ strengths rather than their weaknesses (Avolio et al., 2009); ethical leadership refers to the pursuit of ethical goals and the empowerment of an organization’s members (Gallagher and Tschudin, 2010); mindful leadership refers to a leadership style that focuses on the present moment, acknowledging feelings and emotions and keeping them under control, especially in times of stress; mindful leadership refers to full awareness of people’s presence and your (the leader’s) effect on them (George, 2012; Herold, 2013); benevolent leadership refers to leaders who consider subordinates family members and show an interest in their welfare in their working as well as their private lives (Wang and Cheng, 2010); and decent leadership refers to the previous integrated concepts of leadership (sustainable, servant, authentic, ethical, mindful leadership, and benevolent) and includes also diversity management leadership related to the asset of different resources in an organization (Di Fabio, in press).

### Entrepreneurial Leadership

Entrepreneurial leadership is a distinctive style of leadership that can be present in organizations of any size, type, or age. The literature review revealed that entrepreneurial leadership has been studied by many authors. According to Cunningham and Lischeron (1991), entrepreneurial leadership involves setting clear goals, creating opportunities, empowering people, promoting mutual awareness, and awareness of the organization, and developing a sound human resource system. Ireland et al. (2003) maintain that entrepreneurial leadership is the ability to influence others to manage resources strategically in order to promote opportunity-seeking and advantage-seeking behaviors. Gupta et al. (2004) define entrepreneurial leadership as leadership that creates visionary scenarios that can be used to mobilize and commit subordinates to the discovery and exploitation of strategic value creation. According to Thornberry (2006), leadership in general requires passion, vision, focus, and the ability to inspire others. Entrepreneurial leadership requires all these qualities plus a mindset and skill set that can help entrepreneurial leaders identify, develop, and exploit new business opportunities. Surie and Ashley (2008) define good leadership as the ability to sustain innovation and adaptation in rapidly changing and uncertain environments.

A more recent definition is that entrepreneurial leadership entails influencing and directing the performance of group members toward the achievement of organizational goals that involve recognizing and exploiting entrepreneurial opportunities (Renko et al., 2015). With its explicit focus on the achievement of entrepreneurial goals, this definition can be seen as similar to, yet different from, previous definitions of entrepreneurial leadership.

### Professionalism

Ducheny et al. (1997, p. 89) define professional development as “an ongoing process through which an individual derives a cohesive sense of professional identity by integrating the broadbased knowledge, skills, and attitudes within psychology with one’s values and interests.”

Psychology is not alone in its attempt to explain professional development and professionalism. Epstein and Hundert (2002) considered comparable issues in the training of physicians (e.g., interpersonal functioning and critical thinking; Elman et al., 2005). They focused on “the habitual and judicious use of communication, knowledge, technical skills, clinical reasoning, emotions, values, and reflection in daily practice for the benefit of the individual and community being served” (p. 226). They also considered the acquisition and use of knowledge, integrative aspects of care, building therapeutic relationships, context, development, and “habits of mind.” This last concept encompasses observation of one’s own thinking, emotions, and techniques; attentiveness; critical curiosity; recognition of and response to cognitive and emotional biases; and a willingness to acknowledge and correct errors (p. 230). Attention to professionalism in medicine has burgeoned, and, according to Lundberg (2004), the essential elements of professionalism are self-governance and self-regulation (Elman et al., 2005).

The development of professionalism is the process of acquiring, expanding, refining, and sustaining knowledge, proficiency, skill, and qualifications for competent professional functioning that result in professionalism. This process comprises (a) the internal tasks of clarifying professional objectives, crystallizing professional identity, increasing self-awareness and confidence, and sharpening reasoning, thinking, reflecting, and judgment, and (b) the social/contextual dimension of enhancing interpersonal aspects of professional functioning and broadening professional autonomy (Elman et al., 2005). Professionalism is a necessary component of all aspects of psychologists’ functioning, but it is not a substitute for other factors that contribute to specific competencies (e.g., technical training and clinical and research experience). Professionalism may be conceptualized more as an underlying capacity or capability than a specific skill or competency; some key components of professionalism are self-knowledge, self-assessment, and self-care (Elman et al., 2005).

### Toward a Unified Theoretical and Empirically Based Integrated Framework for Studying High Entrepreneurship, Leadership, and Professionalism

Because flexibility is increasingly required in the world of work, and workers are increasingly called on to deal with challenges and to have more complex skills (Di Fabio, 2016b; Di Fabio and Blustein, 2016a,b; Di Fabio et al., 2016a), it is important to look at entrepreneurship, leadership, and professionalism as three integrated aspects of the successful worker.

In the development of this model, we first started focusing our attention on the construct of entrepreneurial leadership (Renko et al., 2012) that can be conceptualized as the ability to influence others to manage resources strategically in order to promote opportunity-seeking and advantage-seeking behaviors (Ireland et al., 2003) and that involves setting clear goals, creating opportunities, empowering people, promoting mutual awareness and awareness of the organization (Cunningham and Lischeron, 1991). In this integrated background, already existing in the literature, we attempted to enrich this model with the construct of professionalism; because in our point of view, a successful worker need, not only the ability of creating visionary scenarios helpful for the discovery and exploitation of strategic value creation (Gupta et al., 2004), but also a professional identity based on knowledge, skills, and attitudes.

We also considered Kanter’s (1989) theory on the connection between careers and economic, social, and political issues, and also Chan et al’s. (2012) proposal of a person-centered framework for conceptualizing subjective careers. In an increasingly boundaryless work environment, these authors recognized entrepreneurship, leadership, and professionalism (ELP) as three key dimensions of subjective career space.

Because Kanter (1989) conceptualizes the constructs of entrepreneurship, leadership, and professionalism separately, we concentrated more on the work of Chan et al. (2012), which conceptualizes these three variables with the focus on motivation, intentions, and efficacy. In Chan et al’s. (2012) scale, the ELP career aspiration questionnaire, these variables are conceptualized separately; indeed, in our proposed model too, motivation, intentions, and efficacy are conceptualized as three aspects of each dimension analyzed. Entrepreneurship, leadership, and professionalism are thus three integrated variables, and each includes the three aspects of motivation, intentions, and efficacy.

In our point of view we consider the importance of developing a brief and easily administered measure that assesses entrepreneurship, leadership, and professionalism in a complex manner that entertains these three crucial aspects for career success as three integrated parts of a career success model and that can be used both in organizational contexts and with students samples. In this paper we propose a new, self-report instrument for measuring these three variables, considering them in a integrative way, because of the links of motivation, intentions, and efficacy that are present in each of these constructs. The main novelty of this study primarily refers to motivation, intentions, and efficacy that are considered together in each dimension of entrepreneurship, leadership, and professionalism; besides this new scale, directly inspired by the criteria of briefness, capability of being used with different kinds of populations (workers, students, trainers, and trainees) and usefulness for building one’s own personal and professional path, could be something important for training and practice.

### Rationale for the Study

The importance of the following three competencies needed to help people construct careers, shape their identities, and design successful careers and lives was stressed: leadership, entrepreneurship, and professionalism.

This article supports the idea that greater flexibility and an integrated entrepreneurship leadership and professionalism model are needed by people to help them face the challenges and precariousness in the contemporary world of work. In fact, in today’s rapidly changing world of work, a career is more than just a job (Savickas, 2011; Guichard, 2013); it is analogous to a vocation, and it is becoming increasingly important to develop the competences needed to deal with career-life related transitions in the 21st century (Boyatzis et al., 2002, 2015; Boyatzis, 2008; Boyatzis and Saatcioglu, 2008; Boyatzis, 2009; Camuffo et al., 2012; Gerli et al., 2015). The main aim of this study was to support career constructing and life design/life meaning-related interventions and preventive actions (Di Fabio and Bernaud, 2008; Di Fabio and Kenny, 2012; Bernaud, 2013; Bernaud et al., 2015; Di Fabio, 2015; Di Fabio and Palazzeschi, 2015). More particularly, it is hoped that the new measure presented here will increase people’s awareness of key competencies such as entrepreneurship, leadership, and professionalism. The results should help them reflect on the level of these competencies in themselves and also help them deal with life work challenges as these challenges manifest in their career-life stories.

The present study thus examined the psychometric properties of a new measure for assessing these important career dimensions in the HELP questionnaire (HELP-Q). Particularly, we expect significant correlations of the HELP-Q constructs with the other constructs analyzed in the present study because we hypothesize a link between constructs related to self-competencies, self-awareness, and positive attitudes, particularly between scales that intend to assess similar constructs.

## Materials and Methods

### Preliminary Study

A measure consisting of 54 items was developed. In the first phase of the study, we tested the factor structure of the preliminary version of the HELP-Q scale. A convenience sample of 97 students (81 women, 16 men) with a mean age of 24,31 (*SD* = 2.70) enrolled in various psychology courses at the University of Florence and 107 employees (61 women, 46 men) with a mean age of 48,75 (*SD* = 9,66) completed this preliminary version. Exploratory factor analysis (EFA) was used to verify the factor structure of the scale. Velicer’s minimum average partial (MAP) criterion and the inspection of the scree plot suggested a three-factor solution for the measure. In order to obtain a clear, robust factor solution—and on the basis of the theoretical principles and the factor analysis criteria—we eliminated the items with commonalities under 0.30 and duly reached a version with nine items.

### Participants and Procedure

The study participants were 65 workers (36.9% male, 63.1% female) with a mean age of 29.78 years (*SD* = 9.03) and 66 students (34.8% male, 65.2% female) with a mean age of 23.08 years (*SD* = 3.19). The total sample thus numbered 131 subjects (35.9% male, 64.1% female with a mean age of 26.51 years) (*SD* = 7.15).

Significant differences emerged with respect to gender in the Entrepreneurship (E) subscale (χ^2^ = 32.45, *p* = 0.01), in the Leadership (L) subscale (χ^2^ = 24.89, *p* = 0.01), and in the HELP-Q total score (χ^2^ = 49.53, *p* = 0.01), but not in the Professionalism (P) subscale (χ^2^ = 10.91, *p* = 0.282).

The participants first completed the HELP-Q and then, in order to assess aspects of the concurrent validity of this new mirror measure, completed other instruments that assessed similar as well as different constructs. All the instruments were administered in accordance with the norms regarding the privacy and anonymity of participants.

The questionnaires were administered according to the laws of privacy and informed consent of the Italian law (Law Decree DL-196/2003). The participants were told also that they could withdraw from the study at any time and that there would be no payment for participating. Regarding ethical standards for research, the study followed procedures consistent with the latest version of the Declaration of Helsinki revised in Fortaleza (World Medical Association [WMA], 2013).

### Instruments

#### High Entrepreneurship, Leadership, Professionalism – Questionnaire (HELP-Q; Di Fabio, Bucci and Gori, 2016)

The HELP-Q is a short, integrated scale that assesses the principal dimensions of entrepreneurship (E), leadership (L), and professionalism (P) in terms of the areas of motivations, intentions, and efficacies. This scale consists of nine items, three for each area, on a 5-point Likert-type scale (1 = *not at all*, 2 = a little, 3 = *somewhat*, 4 = *much*, 5 = *a great deal*) that helps build a profile of ELP in relation to the three areas.

#### Intrapreneurial Self-Capital Scale (ISCS; Di Fabio, 2014b)

Intrapreneurial self-capital is defined as a core of individual intrapreneurial resources used to cope with career and life construction challenges and includes dimensions of core self-evaluation, hardiness, creative self-efficacy, resilience, goal mastery, decisiveness, and vigilance (Di Fabio, 2014b). The ISCS was developed by Di Fabio (2014b) to measure this new construct of intrapreneurial self-capital. It uses a 5-point Likert-type scale (1 = *strongly disagree*, 2 = *disagree*, 3 = *neither agree nor disagree*, 4 = *agree*, 5 = *strongly agree*) and consists of 28 items (e.g., “I am able to deal with most of my problems,” “I am able to improve the ideas produced by others,” “I am able to achieve objectives despite obstacles,” “One of my goals in training is to learn as much as I can”). In the present study, we used the Italian version of the ISCS, which showed good internal consistency (α = 0.86).

#### Psychological Capital Questionnaire (PCQ; Luthans et al., 2007)

Psychological capital is a new construct measured with the Psychological Capital Questionnaire (PCQ). This measure consists of 12 items with four subscales: (1) hope (e.g., “I feel confident contributing to discussions about the organization’s management”); (2) efficacy (e.g., “I can think of many ways to reach my current work goals”); (3) optimism (e.g., “I’m optimistic about what will happen to me in the future pertaining to work”); and (4) resilience (e.g., “I usually take stressful things at work in stride”). Each of these subscales consists of six items with response options on a six-point Likert scale ranging from 1 (*strongly disagree*) to 6 (*strongly agree*). The PCQ has good psychometric properties also in its Italian version, which was used in the present study (hope = 0.75; efficacy = 0.78; resilience = 0.70; optimism = 0.77; the overall scale = 0.81; Alessandri et al., 2015).

#### Proactive Personality Scale (PPS; Bateman and Crant, 1993)

This scale consists of 17 items on a seven-point Likert scale ranging from 1 (*strongly disagree*) to 7 (*strongly agree*). Such items include: “I excel at identifying opportunities” and “No matter what the odds, if I believe in something, I will make it happen.” The items are summed to arrive at a proactive personality score. The unidimensionality of the scale is supported by factor analysis and reliability across three samples (ranging from 0.87 to 0.89) (Bateman and Crant, 1993). The PPS has good psychometric properties also in its Italian version, which was used in the present study (*a* = 0.81) (Di Fabio and Gori, 2016c).

#### Rosenberg Self-Esteem Scale (RSES; Rosenberg, 1965)

The Rosenberg Self-Esteem Scale (RSES) is a 10-item scale for assessing global self-esteem with the items answered on a 4-point Likert scale ranging from *strongly agree* to *strongly disagree*. Examples of the items: “On the whole, I am satisfied with myself,” “I have a positive attitude toward myself.” The psychometric properties of the RSES have been reported as good in several studies (Corwyn, 2000). In this study, the Italian version of the scale was used (*a* = 0.84) (Prezza et al., 1997).

#### Satisfaction With Life Scale (SWLS; Diener et al., 1985)

The Satisfaction With Life Scale (SWLS) is a self-report instrument that measures global life satisfaction. It consists of five items with responses on a 7-point Likert scale with higher values corresponding to a higher degree of life satisfaction. Examples of the items: “I am satisfied with my life,” “The conditions of my life are excellent.” The psychometric properties of the SWLS are good, with different studies reporting a unidimensional structure of the measure (Diener et al., 1985). In this study, the Italian version of the scale was used (*a* = 0.85) (Di Fabio and Gori, 2015).

### Data Analysis

Factor analysis was used to identify the underlying dimensions of the HELP career construction questionnaire using various criteria for item selection according to the number of selected factors and item factor loadings. In order to verify some assumptions, we used Bartlett’s Test of Sphericity and Kaiser-Meyer-Olkin’s (KMO) Measure of Sampling Adequacy to determine whether the items were significantly correlated and shared sufficient variance to justify factor extraction. Principal axis factoring was selected as the method of factor extraction with oblique rotation (promax criterion) to obtain a simple structure as there was no theoretical assumption to suggest that the factors were independent of each other. Eigenvalues greater than 1, the Kaiser criterion, and the scree test were checked for agreement (Giannini et al., 2011), and their salience was determined by applying the following criteria: a) a factor loading of at least 0.3 on the primary factor, ensuring a high degree of association between the item and the factor; (b) a difference of 0.3 between loading on the primary factor and loading on other factors, ensuring that each item could be considered salient to one factor when an item was loading simultaneously on two factors; (c) a minimum of three items for each factor thus ensuring meaningful interpretation of stable factors (Craparo et al., 2015). The standard Pearson correlation coefficient was used to investigate to what extent the factor scores were intercorrelated. The reliability of the scales of the questionnaire was calculated by means of the alpha coefficient. A confirmatory factor analysis (CFA) was performed using maximum likelihood (ML) estimation procedures. To assess the closeness of the hypothetical model to the empirical data statistically, multiple goodness-of-fit indices were used, including the ratio of the chi-square to degrees of freedom (χ2/*df*), the Non-Normed Fit Index (NNFI), the Comparative Fit Index (CFI), the Standardized Root Mean Square Residual (SRMR), and the Root Mean Square Error of Approximation (RMSEA). Bentler and Bonnet (1980) contend that values greater than 0.90 indicate acceptable fit for the NNFI while values below 0.90 indicate a need to respecify the model; Hu and Bentler (1999) contend that CFI values greater than 0.90 are needed; and Byrne (1994) contends that a cutoff of 0.93 should indicate a good fit. SRMR and RMSEA values less than 0.08 (Browne and Cudeck, 1993), and ideally equal to or less than 0.05, are interpreted as indicating models that fit well (Steiger, 1990; Schermelleh-Engel et al., 2003; Giannini et al., 2011; Gori et al., 2015). Several aspects of concurrent validity were verified using Pearson’s *r* coefficient.

## Results

An examination of the scree plot (Cattell, 1966), and the percentage of variance accounted for, revealed that as many as three factors should be retained for rotation. The adequacy of the sample was measured as 0.72, *p* = 0.001 in terms of the Kaiser-Meyer-Olkin test (KMO) (Field, 2009). The exploratory factor analysis (EFA) (promax rotation) showed a factor structure with three principal dimensions (eigenvalues > 1; 3.41, 2.73, 1.33) with 83.01% of total variance explained.

Factor 1 (Leadership), with three items loading above 0.80, had an eigenvalue of 4.41 and accounted for 37.88% of the total variance explained. Factor 2 (Entrepreneurial), with three items loading above 0.80, had an eigenvalue of 2.73 and accounted for 30.32% of the total variance explained. Factor 3 (Professionalism), with three items loading above 0.60, had an eigenvalue of 1.33 and accounted for 14.81% of the total variance explained. The factor structure matrix shows the three independent factors of the questionnaire (**Table 1**).

**Table 1:** Exploratory factor analysis (EFA) results (principal axis factoring method, promax rotation).


**Original item number**	**Factor**		
	**Leadership**	**Entrepreneurship**	**Professionalism**
HELP item 5	0.940		
HELP item 8	0.932		
HELP item 2	0.928		
HELP item 6		0.956	
HELP item 9		0.870	
HELP item 3		0.827	
HELP item 1			0.937
HELP item 4			0.848
HELP item 7			0.840

*Extraction method: principal axis factoring. Rotation method: promax with Kaiser normalization.*

The goodness-of-fit indices of the three factor model showed a good fit of the model to the data. Although the chi-square was significant, the other goodness-of-fit indices showed satisfactory and good values (*x*^2^/df = 3.13, *p* < 0.001; CFI = 0.95; NNFI = 0.93; SRMR = 0.06; RMSEA = 0.11) (**Table 2**).

**Table 2:** Fit indexes for the three-factorial models.

Sample	χ2/*df*	CFI	NNFI	SRMR	RMSEA
One factor model	18.29	0.67	0.65	0.21	0.24
Two factors model	11.7	0.77	0.74	0.15	0.19
Three factors model	3.13	0.95	0.93	0.06	0.11

*CFI, Comparative Fit Index; NNFI, Non-Normed Fit Index; SRMR, Standardized Root Mean Square Residual; RMSEA, Root Mean Square Error of Approximation.*

The reliability of the scales, calculated using Cronbach’s alpha coefficient, indicated good values of internal consistency (Leadership, *a* = 0.92; Entrepreneurship, *a* = 0.92; Professionalism, *a* = 0.90; and the overall scale = 0.77).

The HELP career construction questionnaire (HELP-Q; see **Appendix**) showed strong correlations with the measures used to assess aspects of concurrent validity (**Table 3**). In particular, the relationship between the HELP-Q and these measures indicates that the HELP-Q is a measure strictly related to individual intrapreneurial resources (Intrapreneurial Self-Capital Scale), to self-capital (Psychological Self-Capital Scale), to proactivity (Proactivity Scale), to self-esteem (Rosenberg Self-Esteem Scale), and to entrepreneurship, professionalism, and leadership (Entrepreneurship, Leadership, and Professionalism Career Aspiration Questionnaire), while it seems not to be significantly related to life satisfaction (Satisfaction With Life Scale).

**Table 3:** Summary of the correlation between the two parts of the ACS and ISC, PSC, F, IOS, PSR, MSPSS.

	ISCS	PSCS	PPS	RSES	SWLS	ELP CAQ
(1) Leadership	0.606^∗∗^	0.452^∗∗^	0.543^∗∗^	0.412^∗∗^	0.155	0.626^∗∗^
(2) Entrepreneurship	0.445^∗∗^	0.399^∗∗^	0.573^∗∗^	0.163	0.109	0.546^∗∗^
(3) Professionalism	0.467^∗∗^	0.484^∗∗^	0.636^∗∗^	0.351^∗^	0.005	0.539^∗∗^
HELP total score	0.598^∗∗^	0.518^∗∗^	0.680^∗∗^	0.360^∗∗^	0.113	0.672^∗∗^


*^∗^p < 0.05, ^∗∗^p < 0.01. ISCS, Intrapreneurial Self-Capital Scale; PSCS, Psychological Self-Capital Scale; PPS, Proactive Personality Scale; RSES, Rosenberg Self-Esteem Scale; SWLS, Satisfaction With Life Scale; ELP, Entrepreneurship, Leadership, and Professionalism Career Aspiration Questionnaire.*

## Discussion

In the contemporary world of work, because of the financial crisis and as consequences the use of new policies, workers need to develop specific characteristics to help them adapt to the new, complex scenarios characterized by high degrees of uncertainty (Di Fabio et al., 2013; Di Fabio and Bucci, 2015, 2016; Di Fabio and Gori, 2016a,b; Di Fabio and Palazzeschi, 2016). Personal characteristics such as entrepreneurship, leadership, and professionalism can help make people more flexible and help them evolve in their careers and in their lives.

In this article, along with the further issue of integrating entrepreneurship research and theory into the more established traditions of leadership and management and with the integration of professionalism, we have discussed a new integrated model of entrepreneurship, leadership, and professionalism. It is hoped that the model will assist the design of future research in these areas by highlighting the common trends and common threads of thought that underlie them. A comprehensive model of these constructs may also help people grow in their careers.

The study also set out to develop a new measure for assessing entrepreneurship, leadership, and professionalism, as well as the psychometric properties of the measure. The exploratory factor analysis revealed a good structure with three principal dimensions: entrepreneurship, leadership, and professionalism. Despite the reduced number of items, each dimension yielded a good Cronbach’s alpha (Nunnally and Bernstein, 1994).

Confirmatory factor analysis also revealed a good fit of the three factor model to the empirical data, as indicated by the fit indices. In particular, for the three factor model, the CFA results showed that the SRMS were 0.06 indicating a good fit (Browne and Cudeck, 1993; MacCallum et al., 1996). The NNFI index indicated an acceptable fit (Byrne, 1994) and the TLI index a satisfactory fit (Bentler and Bonnet, 1980). Regarding the RMSEA result, while some researchers (Browne and Cudeck, 1993; MacCallum et al., 1996) have used 0.01, 0.05, and 0.08 to indicate excellent, good, and mediocre fit, respectively, other researchers consider 0.10 a reasonable cutoff for good vs. poor fitting models if the other indices used to verify the model fit are good or acceptable, which was the case in this study (Kenny et al., 2014).

Correlations between the HELP-Q and the measures used to verify aspects of concurrent validity showed good values: all the relationships between the variables under investigation were in the right direction and with the right significance.

Regarding the limitations of the study, the major limitation was the small number of participants, although this number met the sufficient criterion (Winter et al., 2009). Future studies on this topic should nevertheless use larger samples to provide information about potential gender differences and also take advantage of cross-cultural studies. An additional limitation of this study concerns the absence of an appropriate evaluation of nomological validity; future research should examine various aspects of the nomological associations of the constructs in the HELP-Q.

Despite these limitations, the results of the study are promising and indicate that the HELP-Q model can be of use to those working in guidance, career counseling, career planning, career life construction, human resources, and organizational development. Those who use the model to identify preventive individual resources in terms of high entrepreneurship, leadership, and professionalism will be better able to handle and succeed in a constantly changing labor market. The model can also be of help in designing one’s own future, in creating one’s own opportunities, in reinforcing adaptability skills, in maintaining employability and proactivity, and in constructing one’s self, identity, and life (Savickas, 2011; Guichard, 2013). The integrated HELP model thus underlines the value of personal entrepreneurship, leadership, and professionalism as an integrated preventive core of competencies that can flexibly, adaptively, and proactively build one’s own personal and professional path (Di Fabio, 2014a; Di Fabio et al., 2016a) and ensure success (Akhouri and Sharma, 2009). It can also help people cope with the new reality of the world of work (Arenas et al., 2015; Giorgi et al., 2015, 2016; Mucci et al., 2016) and preserve their individual potentialities and talents (Di Fabio, 2006, 2011; Di Fabio and Palazzeschi, 2008; Kenny and Hage, 2009; Blustein, 2011; Guichard, 2013). The value of the HELP model is that it helps prevent potential career decision-making problems or failures (Sartori et al., 2013, 2015; Ceschi et al., 2014) rather than focusing just on remediation (Di Fabio and Palazzeschi, 2012; Di Fabio et al., 2012, 2013). This new model calls for timeous actions to enhance the mentioned set of competencies at school or at university from a primary preventive perspective. The model calls also for a high level of continuing education as the study results suggest that the HELP-Q can help people prevent future lifelong career problems. Assessing the level of these competencies can also help people develop these competences when they are low. The HELP model can be used also to devise career services, career counseling goals, and new managerial practices in organizational and community contexts to help people cope with the complex career issues of the 21st century.

## Conclusion

The study showed that the HELP-Q is a model that can be profitably used to help people adjust to change and manage their life and work opportunities.

## Ethics Statement

The administration adhered to the requirements of privacy and informed consent in Italian law (Law Decree DL-196/2003) and the ethical standards for research of the Declaration of Helsinki revised in Fortaleza (World Medical Association [WMA], 2013), followed and approved by the Department of Education and Psychology of the University of Florence (Italy).

The administration adhered to the requirements of privacy in Italian law (Law Decree DL-196/2003) and informed consent was recollected for each participant.

## Author Contributions

AD and AG conceptualized the study, chose measures, designed the scale. OB helped in the collection of the data. AD and AG analyzed the data and wrote the methods and results. Then all authors wrote the paper together and read and revised the manuscript several times.

## Conflict of Interest Statement

The authors declare that the research was conducted in the absence of any commercial or financial relationships that could be construed as a potential conflict of interest.

## Appendix

**High Entrepreneurship, Leadership, Professionalism – Questionnaire (HELP-Q)**

**Annamaria Di Fabio, Ornella Bucci, and Alessio Gori**

**Table:** Please read each statement carefully and indicate the degree to which you recognize yourself in it by choosing the appropriate number based on the following scale.

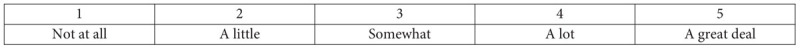

**Table:** (1) My main professional goal is to: (Il mio principale obiettivo professionale è quello di)

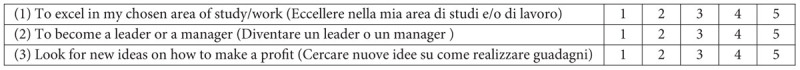

**Table:** (2) To what extent do I feel able to: (In che misura mi sento in grado di):

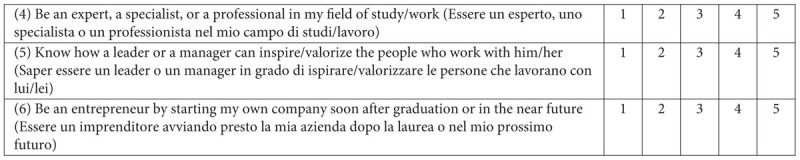

**Table:** (3) To what extent do I feel able to: (In che misura mi sento in grado di):
